# *CONSTANS* is a photoperiod regulated activator of flowering in sorghum

**DOI:** 10.1186/1471-2229-14-148

**Published:** 2014-05-28

**Authors:** Shanshan Yang, Brock D Weers, Daryl T Morishige, John E Mullet

**Affiliations:** 1 Department of Biochemistry and Biophysics, Texas A&M University, College Station, TX 77843-2128, USA

**Keywords:** Photoperiod, Sorghum, Flowering time, QTL, CONSTANS, PRR37

## Abstract

**Background:**

Sorghum genotypes used for grain production in temperate regions are photoperiod insensitive and flower early avoiding adverse environments during the reproductive phase. In contrast, energy sorghum hybrids are highly photoperiod sensitive with extended vegetative phases in long days, resulting in enhanced biomass accumulation. SbPRR37 and SbGHD7 contribute to photoperiod sensitivity in sorghum by repressing expression of *SbEHD1* and *FT-*like genes, thereby delaying flowering in long days with minimal influence in short days (PNAS_108:16469-16474, 2011; Plant Genome_in press, 2014). The GIGANTEA (GI)-CONSTANS (CO)-FLOWERING LOCUS T (FT) pathway regulates flowering time in Arabidopsis and the grasses (J Exp Bot_62:2453-2463, 2011). In long day flowering plants, such as Arabidopsis and barley, CONSTANS activates *FT* expression and flowering in long days. In rice, a short day flowering plant, Hd1, the ortholog of CONSTANS, activates flowering in short days and represses flowering in long days.

**Results:**

Quantitative trait loci (QTL) that modify flowering time in sorghum were identified by screening Recombinant Inbred Lines (RILs) derived from BTx642 and Tx7000 in long days, short days, and under field conditions. Analysis of the flowering time QTL on SBI-10 revealed that BTx642 encodes a recessive *CONSTANS* allele containing a His106Tyr substitution in B-box 2 known to inactivate CONSTANS in *Arabidopsis thaliana*. Genetic analysis characterized sorghum CONSTANS as a floral activator that promotes flowering by inducing the expression of *EARLY HEADING DATE 1* (*SbEHD1*) and sorghum orthologs of the maize FT genes *ZCN8* (*SbCN8*) and *ZCN12* (*SbCN12*). The floral repressor *PSEUDORESPONSE REGULATOR PROTEIN 37* (PRR37) inhibits sorghum CONSTANS activity and flowering in long days.

**Conclusion:**

Sorghum CONSTANS is an activator of flowering that is repressed post-transcriptionally in long days by the floral inhibitor PRR37, contributing to photoperiod sensitive flowering in *Sorghum bicolor*, a short day plant.

## Background

Optimal regulation of the timing of floral transition is critically important for reproductive success and crop yield. The C4 grass *Sorghum bicolor* is widely adapted and grown as an annual crop from 0 to >40 degrees N/S latitude. Sorghum crops have been selected for a range of flowering times depending on growing location and use as a source of grain, sugar, forage, or biomass [1-3]. Grain sorghum is generally selected for early flowering (60–80 days) to enhance grain yield stability by avoiding drought, adverse temperatures, and insect pressure during the reproductive phase. In contrast, energy sorghum hybrids are designed with high photoperiod sensitivity in order to delay flowering and extend the duration of vegetative growth, resulting in more than 2-fold increases in biomass production [3,4]. The stage of plant development, signals from photoperiod, temperature, gibberellins and other factors are integrated to regulate flowering time in sorghum [5].

The genetic architectures of photoperiod-responsive flowering-time regulatory pathways have been characterized in many plants [6-18]. In Arabidopsis, flowering is promoted in long-days (LD) by coincidence of light signaling and circadian clock output, thus allowing the plant to sense and respond to seasonal changes in photoperiod. Clock output to the flowering pathway is mediated in part by *GIGANTEA* (*GI*). GI is regulated by the central clock oscillator comprised of *TIMING OF CAB EXPRESSION 1* (*TOC1*), *CIRCADIAN CLOCK ASSOCIATED 1* (*CCA1*) and *LATE ELONGATED HYPOCOTYL* (*LHY*). In long days, GI activates *CONSTANS* (*CO*) expression in conjunction with *FLAVIN-binding KELCH DOMAIN F BOX PROTEIN1* (*FKF1*) by inducing degradation of CDF1 repressors of *CONSTANS* transcription. CO accumulates in LD due to stabilization mediated by cryptochromes (CRY1/2), phytochrome A (PHYA) and SUPPRESSOR *OF PHYA-105* (*SPA1*) that counteract degradation of CO mediated by phytochrome B (PHYB): *CONSTITUTIVE PHOTOMORPHOGENIC 1*(*COP1*) [6,9]. Increased CO protein levels in long days leads to the activation of *FLOWERING LOCUS T* (*FT*) expression and production of florigen that moves from leaves to shoot apical meristems (SAM) where it binds to FD and induces floral transition.

The GI-CO-FT regulatory pathway identified in Arabidopsis, a long day (LD) plant, is also present in rice, a short day (SD) plant [10]. When rice is exposed to inductive SD, *HEADING DATE1* (*Hd1*), the ortholog of *CO*, activates expression of the *FT*-like gene *Hd3a*, one of two sources of florigen in rice. In non-inductive LD, Hd1 functions as a repressor of *Hd3a* and flowering [19]. Thus, photoperiod sensitivity in rice depends in part on differences in the activity of CO (Hd1) in long days and short days. Two modulators of flowering time unique to grasses were identified in rice: *EARLY HEADING DATE 1* (*EHD1*) [20] and *GRAIN NUMBER, PLANT HEIGHT AND HEADING DATE 7* (*GHD7*) [21,22]. EHD1 activates the expression of *Hd3a* and *RICE FLOWERING LOCUS T1* (*RFT1*), a source of florigen in long days. GHD7 represses flowering by down-regulating expression of *EHD1* and *Hd3a* in LD in rice [23] and *SbEHD1* and *SbCN8* in sorghum [2].

The effect of photoperiod on flowering time varies extensively among and within grass species. Barley and wheat are LD plants, while rice and sorghum are SD plants. Most cultivated maize is photoperiod insensitive therefore plants flower after a set number of degree days; however tropical maize is a photoperiod sensitive short day plant [11]. Sorghum is a short day plant, although grain sorghum is usually photoperiod insensitive, and forage and energy sorghum genotypes exhibit varying degrees of photoperiod sensitivity [3]. More than 40 QTL for flowering time have been identified in sorghum [24]. The *Ma1-Ma4* loci were discovered while breeding for early flowering photoperiod insensitive grain sorghum in the U.S. (1920–1960) [25]. *Ma1* corresponds to *PSEUDORESPONSE REGULATOR PROTEIN 37* (*SbPRR37*), a repressor of flowering in LD [1]. *Ma3* encodes *PHYTOCHROME B* (PhyB), a red-light photoreceptor that plays an important role in photoperiod sensing and repression of flowering [26-28]. *Ma6* encodes SbGhd7, a repressor of *SbEHD1* expression and flowering in long days [2]. *Ma2, Ma4*, and *Ma5* are flowering time loci that enhance photoperiod sensitivity in sorghum [25,29].

CONSTANS (CO) was initially identified as a transcriptional activator of *FT* and flowering in Arabidopsis [30]. CO belongs to a family of transcription factors unique to plants that contain one or two N-terminal zinc finger B-box domains and a C-terminal CCT domain. Two conserved cysteine and histidine amino acids in the Zn finger domain are essential for CO activity [6]. Arabidopsis mutants with amino acid substitutions at these positions have late flowering phenotypes. Extensive gene duplication events have occurred in this gene family, resulting in ~17 CO family members in Arabidopsis, ~16 in rice and ~9 in barley [31,32]. The ortholog of CONSTANS in rice, Hd1, plays a key role in photoperiod regulation of flowering, by activating flowering in SD and repressing flowering in LD [19]. Alleles of *Hd1* account for ~44% of the variation in flowering time observed in cultivated rice [33]. *Hd1* transcript and protein levels are similar in LD and SD, consistent with the finding that Hd1 activity is modulated post-transcriptionally by PHYB [34] and PRR37 [35,36].

## Results

### Identification of flowering time QTL

**Figure 1:**
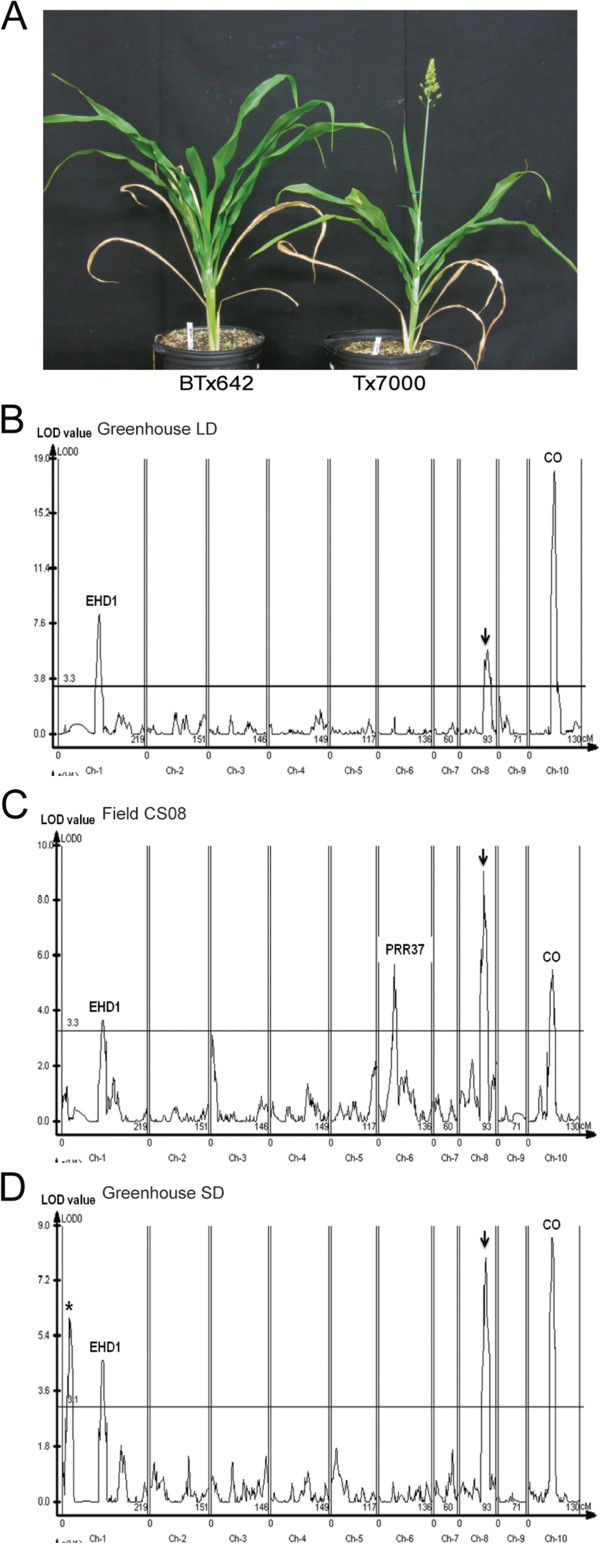
**Genetic basis of flowering time variation in the BTx642/Tx7000 RIL population. (A)**. Flowering time phenotypes of BTx642 and Tx7000 in LD. (Days to flowering for BTx642 and Tx7000 are 103 and 84.) Flowering time QTL identified when RIL population were grown in a LD greenhouse **(B)**, under field conditions in 2008 **(C)** and in a SD greenhouse **(D)**. Permutation tests were carried out to identify 95% confidence thresholds and significant threshold of LOD score is presented as a horizontal red line. Candidate genes associated with main affect QTL are noted above peaks.

Flowering time QTL were mapped in a RIL population derived from a cross of BTx642 and Tx7000, genotypes used in U.S. grain sorghum breeding programs as sources of drought tolerance [37]. A RIL population (n = 90) derived from these genotypes was previously used to map QTL for flowering time and the stay-green drought tolerance trait using a genetic map based on RFLP markers [38]. The genomes of BTx642 and Tx7000 were recently sequenced and analyzed for variation in DNA polymorphisms that distinguish these genotypes [39]. Digital Genotyping was used to create a high-resolution genetic map aligned to the genome sequence based on this RIL population [39,40]. Digital Genotyping identified 1,462 SNP markers segregating in the RIL population and data on recombination frequency was used to create a 1139 cM genetic map spanning the 10 sorghum chromosomes [39]. Flowering time QTL were mapped in this population by phenotyping the RIL population for days to half pollen shed in greenhouses in 14 h long days (LD), 10 h short days (SD), and under field conditions where day length increases following plant emergence in mid-April from 12.6 h to 14.3 h in July. Tx7000 flowered in 73 days and BTx642 flowered approximately 4 days later under field conditions in College Station, Texas. When grown in a greenhouse at constant 14 h day lengths (LD) during the summer, Tx7000 flowered in 84 days and BTx642 flowered ~19 days later (Figure 1A). When Tx7000 and BTx642 were grown in a greenhouse under 10 h day lengths (SD) during the winter, Tx7000 flowered in 54 days whereas BTx642 flowered ~11 days later.

The BTx642/Tx7000 RIL population was grown and assayed for days to flowering under field conditions in 2008–2010, in LD greenhouses in 2009 and 2010, and in a SD greenhouse during the winter of 2011. WinQTL Cartographer was used to identify flowering time QTL using flowering time data collected from each location/year and the genetic map generated by Evans et al. [39]. Three QTL for flowering time were observed in every environment and two additional QTL were identified in only one environment (Table 1).

**Table 1:** Parameters of flowering time QTLs in BTx642/Tx7000 RILs population

**Greenhouse LD (14 h)**							
**QTL**	**Candidate gene**	**Chromosome number**	**Position (cM)**^**a**^	**LOD score**	**Peak coordinate**^**b**^	**Additive effect**^**c**^	**R**^**2d**^
1	*EHD1*	Chr_01	102.7	8.31	22012456- 22012527	-6.25	0.12
2	ND^**e**^	Chr_08	67.9	5.82	50255989- 50256060	-5.02	0.08
3	*CO*	Chr_10	61.7	18.43	13696999- 13697070	-12.69	0.40
**Field LD condition CS08**							
1	*EHD1*	Chr_01	102.7	3.74	22012456- 22012527	-1.09	0.09
2	*PRR37*	Chr_06	42.0	5.71	40201054- 40201125	1.53	0.15
3	ND	Chr_08	60.2	9.09	49290307- 49290378	-1.80	0.26
4	*CO*	Chr_10	59.7	4.11	10080053- 10080126	-1.50	0.16
**Greenhouse SD (10 h)**							
1	ND	Chr_01	16.3	6.00	7208344- 7208415	2.18	0.09
2	*EHD1*	Chr_01	102.7	4.92	22012456- 22012527	-1.80	0.07
3	ND	Chr_08	65.1	7.96	49797259- 49797330	-2.46	0.14
4	*CO*	Chr_10	59.7	8.70	10080053- 10080126	-3.30	0.17

^**a**^Position of likelihood peak (highest LOD score).

^**b**^Peak coordinate: physical coordinate of the likelihood peak.

^**c**^Additive effect: A positive value means the delay of flowering time due to Tx7000 allele. A negative value means the delay of flowering time due to BTx642 allele.

^**d**^R^2^ (coefficient of determination): percentage of phenotypic variance explained by the QTL.

^**e**^ND: Candidate gene is not determined.

Three flowering time QTL were identified when RILs were screened in LD greenhouse conditions (Figure 1B). The QTL on SBI-01 (19.2-22.0 Mbp) explained 12.3% of the phenotypic variance for flowering time in this environment. *SbEHD1*, an activator of flowering in grasses located on SBI-01 (Sb01g019980, 21921315–21925396) was found in a one LOD interval spanning this QTL. *SbEHD1* was previously identified as a floral activator in sorghum based on sequence similarity to rice *EHD1* and observed changes of *SbEHD1* expression in LD compared to SD, consistent with this function [1]. There were no amino acid differences between the SbEhd1 protein sequences from Tx7000 and BTx623. BTx623 is a grain sorghum used extensively for breeding, genetic, and genomic research [40]. However, comparison of SbEhd1 from BTx642 and Tx7000 revealed two amino acid substitutions, Asp144Asn and Thr157Ile (Additional file 1: Table S1). The differences in Ehd1 protein sequences occur in a GARP domain that is highly conserved among *OsEHD1*, *SbEHD1* and *ARABIDOPSIS RESPONSE REGULATOR 1/2* (*ARR1/2*). The *SbEHD1* allele in BTx642 (tentatively designated *Sbehd1-2*) delays flowering in LD and SD relative to Tx7000 (*SbEHD1-1*), consistent with the hypothesis that the amino acid changes in *Sbehd1-2* reduce the activity of this floral activator and explaining why a flowering time QTL was detected in this region of SBI-01.

A flowering time QTL located on SBI-10 (10.1-13.7 Mbp) was observed in all environments and spanned a region that encodes a homolog of *CONSTANS* and *Hd1* (Sb10g010050, 12275128–12276617), an important regulator of flowering time in Arabidopsis and rice, respectively (Figure 1B-D). The QTL spanning the sorghum homolog of *CONSTANS* explained ~40% of the variance in flowering time in LD greenhouses, and 16-17% when plants were grown in the field or SD greenhouses (Table 1). A flowering time QTL located on SBI-08 (48.1-50.3 Mbp) was observed in LD, SD and under field conditions. This QTL explained 8-14% of the phenotypic variance in LD and SD and 18-22% of the variance in field environments. Additional analysis will be required to identify the gene corresponding to this flowering time QTL. A QTL located at the end of SBI-01(~7.2 Mbp) was observed only when the BTx642/Tx7000 RIL population was grown in the SD greenhouse (Figure 1D). A QTL on SBI-06 (~40.2 Mbp) explaining ~15% of the variance in flowering time was identified when RILs were grown in the field (Figure 1C). *SbPRR37 (Ma1)*, a repressor of flowering in LD, was located in the flowering time QTL on SBI-06. Sequence analysis showed that BTx642 encodes *Sbprr37-1* and a truncated version of PRR37, and that Tx7000 contains *Sbprr37-2,* encoding a full-length version of PRR37 containing a Lys162Asn change in the pseudo-response regulator domain [1]. Genotypes containing *Sbprr37-2* flowered later than genotypes encoding *Sbprr37-1* (null) under field conditions, indicating that *Sbprr37-2* is an active but weak allele of *SbPRR37*. This conclusion is consistent with analysis of a flowering time QTL aligned to PRR37 identified in a RIL population derived from crossing the genotypes Rio and BTx623 [41]. Sequence analysis of *SbPRR37* alleles showed that Rio encodes *Sbprr37-2* and BTx623 contains *Sbprr37-3*, a null allele [1]. The *Ma1* allele from Rio (*Sbprr37-2*) delayed flowering relative to BTx623 in field conditions in College Station in a manner similar to the delayed flowering attributed to the same allele in Tx7000 compared to BTx642, which encodes the null allele *Sbprr37-1*.

### Identification of sorghum CONSTANS

**Figure 2:**
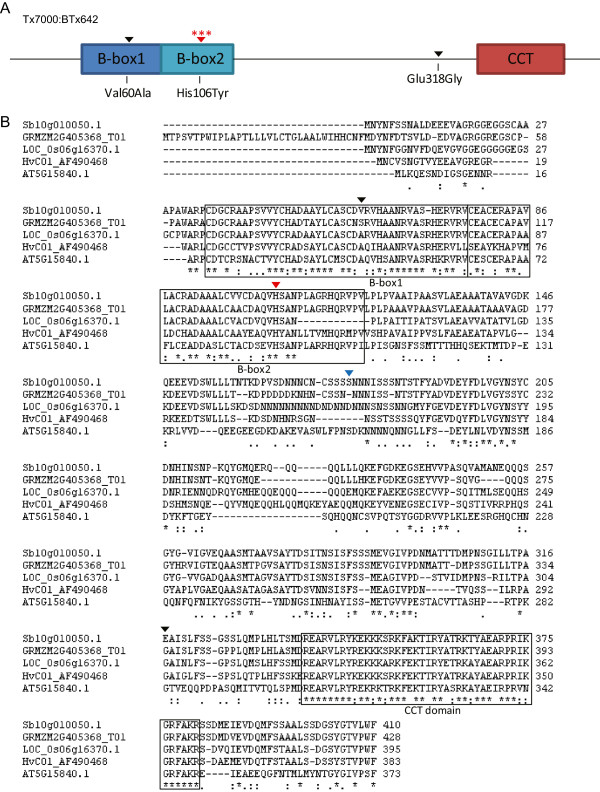
**Multiple alignment analysis of CONSTANS homologs. A**. Protein structure of SbCO showing domains characteristic of CONSTANS-like gene families: B-box1, B-box2 and CCT domain are boxed. Red asterisks above the His106Tyr mutation indicate that this functional mutation was also identified in rice and Arabidopsis. **B**. Multiple sequence alignments of CO homologs from sorghum (Sb10g010050, SbCO), maize (GRMZM2G405368_T01, conz1), rice (Os06g16370, OsHd1), barley (AF490468, HvCO1) and Arabidopsis (AT5G15850, AtCO). The sorghum sequence used for alignment was derived from BTx623 (*SbCO-1*). Protein residues conserved among all 5 species are underscored by asterisks. Amino acid residues underscored by a colon indicate residues of strongly conserved properties, while residues underscored by a period indicate residues with more weakly similar properties. One amino acid substitution distinguishes BTx623 (*SbCO-1*) and Tx7000 (*SbCO-2*) (marked with blue arrow). Unique amino acid substitutions that distinguish BTx623 and BTx642 (*Sbco-3*) are marked with black arrows (tolerant) and a red arrow (intolerant).

The hypothesis that the flowering time QTL on SBI-10 was caused by alleles of *CONSTANS/Hd1* was investigated further through gene sequence alignment and analysis of colinearity. The amino acid sequence of rice Hd1 was used to identify homologs in sorghum, maize, barley and Arabidopsis using data from Phytozome v9.1 [42]. Sb10g010050 (score = 71.9), GRMZM2G405368_T01 (score = 80.7), AF490468 (score = 63.2) and AT5G15850 (score = 40.5) had the highest similarity to Hd1 in each species. GRMZM2G405368_T01 and AF490468 were previously identified as the maize *CONSTANS*-like gene, *conz1*[43] and barley *CONSTANS*-like gene, *HvCO1*[36], respectively, while AT5G15850 encodes *CO* in Arabidopsis [30]. Multiple sequence alignment of the CO homologs showed that Sb10g010050 has all of the characteristic protein domains found in CONSTANS-like gene families (Figure 2), including an N-terminal B-box1 (residues 35–76), B-box2 (residues 77–120) domains and a C-terminal CCT domain (residues 339–381).

The sorghum homolog of *CONSTANS* (Sb10g010050) is located on SBI-10 and rice *Hd1* (Os06g16370) is located on the homeologous rice chromosome 6, suggesting that these genes may be orthologs. The sequences of these genes and adjacent sequences in each chromosome were aligned to determine if *SbCO* and *OsHd1* were in a region of gene colinearity. The sorghum sequences flanking Sb10g010050 were downloaded from Phytozome and aligned with sequences from rice chromosome 6 flanking *Hd1* using GEvo [44]. Three genes and *Hd1* were aligned and in the same relative order in a 100 kbp region in the two chromosomes, consistent with the identification of Sb10g010050 as an ortholog of rice *Hd1* (Additional file 2: Figure S1). Therefore, based on sequence similarity and colinearity, Sb10g010050 was designated as an ortholog of rice *Hd1* and a probable ortholog of Arabidopsis *CO* and termed “*SbCO*”.

The hypothesis that the flowering time QTL on SBI-10 was caused by different alleles of *SbCO* in BTx642 and Tx7000 was investigated further by comparing the *SbCO* sequences from these genotypes. The comparison revealed one difference in intron sequence and four differences in the coding region, three of which cause changes in amino acid sequence (Table 2). The amino acid change Val60Ala, occurs in B-box1 (Figure 2, black arrow), a conservative change in amino acid sequence that is expected to be tolerated based on SIFT analysis [45]. The amino acid change Glu318Gly occurs outside the B-boxes and CCT-domain (Figure 2, black arrow) and was also predicted to be tolerated based on SIFT analysis. While the Val60Ala and Glu318Gly changes in protein sequence may not disrupt CO function, it is possible that other aspects of CO could be modified by these differences. The His106Tyr change in BTx642 CO protein sequence located in B-box2 (Figure 2, red arrow) is predicted to disrupt CO function. In the wild type version of *CONSTANS,* His106 is required for zinc coordination and protein activity [6]. The BTx642 allele of *CONSTANS* was designated *Sbco-3* because the Arabidopsis allele *co-3* has the same His106Try substitution that disrupts function [30]. The wild type alleles of *CO* in BTx623 and Tx7000 had identical CO protein sequences except for a Ser177Asn substitution in Tx7000 (Figure 2B, blue arrow), a modification that does not affect the B-boxes or the CCT domain, and is predicted by SIFT to have minimal impact on CO function. Based on this analysis, the *CONSTANS* alleles in BTx623 and Tx7000 were designated as *SbCO-1* and *SbCO-2*, respectively, and the allele in BTx642 as *Sbco-3*. BTx642 (*Sbco-3)* flowers later than Tx7000 (*SbCO-2*) in both long and short days.

**Table 2:** **Characterization of *SbCO *** **alleles from BTx623, Tx7000 and BTx642**

**SNP #**	**1**	**2**	**3**	**4**	**5**	**6**
Location (SBI-10)	12275306	12275331	12275443	12275657	12276109	12276334
Nucleotide variation	T > C	T > G	C > T	G > A	C > T	A > G
Protein modification	Val60Ala	No change	His106Tyr	Ser177Asn	Intron	Glu318Gly
CONSTANS domain	B-box1		B-box2			
SIFT score	tolerant	N/A*	Intolerant	Tolerant	N/A	Tolerant
*SbCO-1* (BTx623)	-	-	-	-	-	-
*SbCO-2* (Tx7000)	-	-	-	+	-	-
*Sbco-3* (BTx642)	+	+	+	+	+	+

*N/A: Not applicable.

### SbCO alleles modulate expression of genes in the flowering time pathway

The influence of *SbCO* alleles on the expression of other genes in the flowering-time regulatory pathway was analyzed to further understand how *SbCO* affects flowering time. RIL105 and RIL112 were identified that differ in alleles of *SbCO* but not at the other main loci that affect flowering time. RIL105 and RIL112 are homozygous for BTx642 alleles for the flowering time QTL on SBI-01 (spanning *Sbehd1-2*), SBI-06 (spanning *Sbprr37-1*), and SBI-08. BTx642 encodes a null allele of *Ma1* (*Sbprr37-1*), a gene that contributes to photoperiod sensitivity. Tx7000 contains a weak allele of *Ma1* (*Sbprr37-2*) that encodes a full-length protein that inhibits flowering based on QTL analysis [1,41]. Therefore, RIL105 and RIL112 were selected for expression studies because both contain DNA from BTx642 on SBI-06 from 0-42 Mbp, ensuring that these genotypes are null for *Ma1* (*Sbprr37-1*). In addition, both RILs encode a null allele of *Ma6* (*Sbghd7-1*) located at the proximal end of SBI-06 [2]. Therefore, comparison of gene expression in RIL105 and RIL112 caused by differences in *SbCO* alleles will not be influenced by *Ma1* or *Ma6* the main determinants of photoperiod sensitivity in sorghum [1,2].

**Figure 3:**
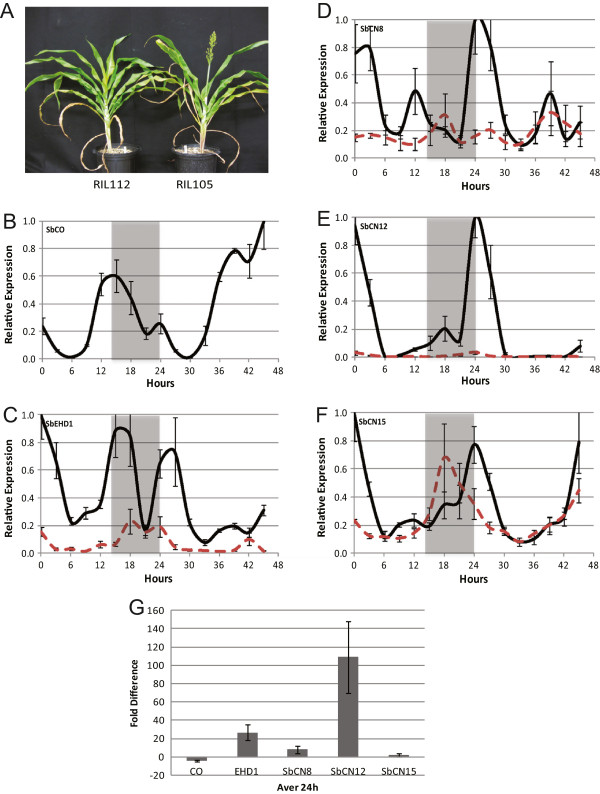
**SbCO promotes flowering by inducing *SbEHD1 *and *FT*-like genes in LD (14 h light/10 h dark). A**. Flowering time phenotype of RIL112 and RIL105. (Days to flowering for RIL112 and RIL105 are 113 and 75.) **B-F**. Relative expression levels of flowering time genes in RIL105 (black solid line) and RIL112 (red dashed line). Gray shading denotes the dark/night portion of each 24 h cycle. The first 24 h covers one light–dark cycle, followed by 24 h of continuous light and temperature (LL). **B**. SbCO. **C**. SbEHD1. **D**. SbCN8. **E**. SbCN12. **F**. SbCN15. **G**. Average fold differences of the first 24 h (light–dark cycle) between the mRNA levels of each gene in RIL105 and RIL112 is plotted. Positive values represent higher expression detected in RIL105. Each expression data point corresponds to three technical replicates within three biological replicates.

When grown in a LD greenhouse, RIL105 (*SbCO-2*) flowered in ~75 days, whereas RIL112 (*Sbco-3*) flowered in ~113 days consistent with the hypothesis that SbCO functions as an activator of flowering (Figure 3A). *SbCO* expression in RIL105 (*SbCO-2*) was analyzed using qRT-PCR during a 24 h LD cycle followed by 24 h of continuous light and temperature (LL). *SbCO* expression decreased at dawn and remained at low levels during most of the day and then increased to a peak in the evening, approximately 15 h after dawn, followed by a decline and second smaller peak at dawn (Figure 3B). The peaks of *SbCO* expression in the evening and near dawn were previously observed in sorghum [1] and for *conz1* in maize [43]. The increase in *SbCO* expression in the evening also occurred in continuous light (LL), consistent with prior studies showing that light and the circadian clock modulate this peak of *CO* expression. The pattern of *SbCO* expression in RIL112 (*Sbco-3*) was similar to RIL105 (*SbCO-2*) although with slightly higher (<2-fold) levels of expression (data not shown).

RIL105 (*SbCO-2*) and RIL112 (*Sbco-3*) were used to analyze how alleles of *CONSTANS* affect expression of other genes in the sorghum flowering time regulatory pathway. Expression of the clock genes *TOC1, LHY* and *GI* were similar in RIL105 and RIL112, indicating that these genes are not affected by *SbCO* alleles as expected for genes upstream of *SbCO* (Additional file 3: Figure S2). In Arabidopsis CO activates flowering by inducing expression of *FT* and in rice Hd1 activates *Hd3a/RFT1*, genes encoding PEBP (phosphatidylethanolamine-binding) domain protein ‘florigens’ that move from the leaf to the shoot apical meristem where they interact with FD and induce floral transition. In rice, two members of the PEBP gene family, *Hd3a* and *RFT1* were identified as encoding florigens [10]. In maize, *ZCN8*, a different member of the PEBP gene family, was identified as a source of florigen [46,47]. In sorghum, *SbCN8* and *SbCN12* are potential sources of florigen because expression of both genes is regulated by photoperiod, modulated by *Ma1* alleles*,* and induction of expression occurs coincident with floral initiation [1]. The sorghum orthologs of maize *ZCN8* (*SbCN8*), *ZCN12* (*SbCN12*) and rice *Hd3a* (*SbCN15*) were identified and qRT-PCR primers specific to each gene were designed to enable analysis of gene expression (Additional file 4: Table S2). No ortholog of *RFT1* is present in the sorghum genome.

In leaves of RIL105 (*SbCO-2*) grown in LD, *SbEHD1* expression was high at dawn and then declined during the day before increasing in the evening approximately 15 h after dawn (Figure 3C, black solid line), with a pattern similar to *SbEHD1* expression in 100 M (*Ma1*) in short days [1]. During the 24 h LD cycle, *SbEHD1* RNA was higher in RIL105 (*SbCO-2*) compared to RIL112 (*Sbco-3*) indicating that CO activates expression of *SbEHD1* (Figure 3C, RIL112 = red dashed line). The average difference in *SbEHD1* RNA level in the two RILs during the 24 h LD cycle was 20-fold (Figure 3G). In leaves of RIL105 (*SbCO-2*) grown in LD, *SbCN8* and *SbCN12* mRNA levels were highest at dawn, then decreased during the day with a second smaller peak of expression approximately 12-18 h after dawn (Figure 3D and E, black solid line). In RIL112 (*Sbco-3*), the same pattern of expression was observed; however, *SbCN8* and *SbCN12* mRNA levels were much lower (Figure 3D and E, red dashed line). Expression of *SbCN8* was ~10-fold higher in RIL105 (*SbCO-2*) compared to RIL 112 (*Sbco-3*) (Figure 3C) and expression of *SbCN12* was ~100-fold higher in RIL105 (*SbCO-2*) compared to RIL112 (*Sbco-3*) (Figure 3D) over a 24 h LD cycle in the *SbCO-2* background (Figure 3G). In contrast, the mRNA level of *SbCN15* (*Hd3a*) was similar in the two genotypes (Figure 3G), although the gene’s peak of expression was at dawn in RIL105 (*SbCO-2*) and at 18 h in RIL112 (*Sbco-3*) (Figure 3F). Together, these results are consistent with the hypothesis that SbCO promotes flowering by inducing expression of *SbEHD1*, *SbCN8,* and *SbCN1*2, with *SbCN12* showing the largest CO-mediated increase in expression in LD.

### Regulation of SbCO floral promoting activity in SD and LD

**Figure 4:**
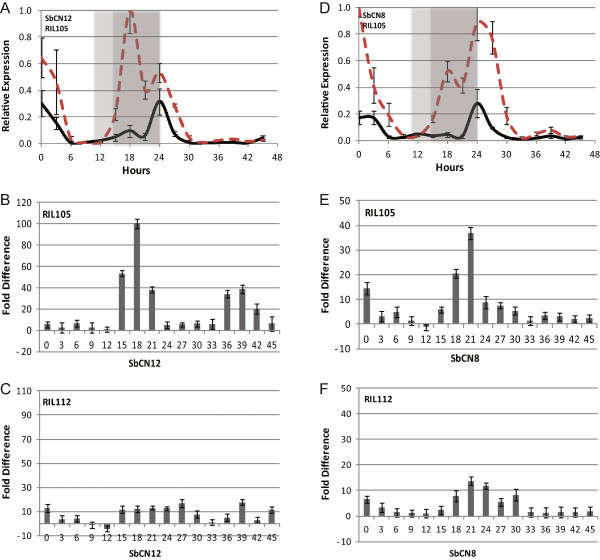
**Relative expression levels and fold differences of *SbCN8 *and *SbCN12 *mRNA in plants (RIL105 or RIL112) grown in LD (14 h light/10 h dark) or SD (10 h light/14 h dark).** Black solid lines represent relative expression in LD and red dashed lines represent relative expression in SD. Positive fold difference values indicates higher mRNA levels detected under SD condition. **A-C**. SbCN12. **D-F**. SbCN8.

Comparison of flowering time and flowering pathway gene expression in RIL105 (*ma1, ma6, CO*) and RIL112 (*ma1, ma6, co-3*) showed that SbCO activates *SbCN8/12* expression and flowering in LD. The next question addressed was whether photoperiod alters SbCO activity in sorghum. When grown in a SD greenhouse, RIL105 (*SbCO-2*) flowered in ~55 days, whereas RIL112 (*Sbco-3*) flowered in ~72 days consistent with the hypothesis that SbCO functions as an activator of flowering in short days in sorghum. A comparison of the relative expression of *SbCO* in SD and LD showed that *SbCO* expression was not altered significantly in response to day-length (Additional file 5: Figure S3). However, differences in the relative ability of SbCO to activate expression of *SbCN12* and *SbCN8* in SD and LD were observed in comparisons of RIL105 (*ma1, ma6, CO*) and RIL112 (*ma1, ma6, co-3*) (Figure 4). In RIL105, *SbCN12* and *SbCN8* had higher expression in SD compared to LD, especially during the night when both genes showed their highest expression (Figure 4A and D; SD = red dashed line, LD = solid line). The difference between *SbCN12* mRNA levels in SD and LD varied depending on time of day, with the largest differences occurring during the night, peaking at 18 h (Figure 4B). A similar pattern was observed for *SbCN8* where expression in SD was 20–40 fold higher during the night in SD, peaking between 18-21 h (Figure 4E). When a comparison of *SbCN8/12* expression in SD/LD was done using RIL112 (*Sbco-3*), ~10-fold differences in expression in SD vs. LD were observed during the night (Figure 4C and F). Taken together, these results indicate that CO has greater activity in SD compared to LD causing up to 10-fold higher expression of *SbCN8/12* during the night in genetic backgrounds that contain null alleles of *Ma1* and *Ma6*.

### Post-transcriptional inhibition of SbCO activity by PRR37 (Ma1)

In sorghum, *Ma1* (*SbPRR37*) increases photoperiod sensitivity by repressing expression of *SbEHD1* and *SbCN8/12*, resulting in delayed flowering in LD but with minimal effect in SD [1]. The ability of SbPRR37 to inhibit expression of *SbCN8* and *SbCN12* could be due to inhibition of SbEHD1 or SbCO, activators of *SbCN8* and *SbCN12* expression*,* and/or by direct inhibition of SbCN8 and SbCN12. A flowering time QTL coincident with *Ma1* was identified in the BTx642/Tx7000 RIL population grown under field conditions in 2008, 2009 and 2010 (e.g. Figure 1C) as well as in Lubbock, Texas (data not shown). This QTL was not observed in SD conditions, as expected, because SbPRR37 has minimal impact on flowering under these conditions. As noted above, BTx642 encodes a null allele *Sbprr37-1*, however, the *Ma1* allele in Tx7000, *Sbprr37-2*, encodes a full-length protein with one amino acid substitution Lys62Asn with sufficient activity to delay flowering time under field conditions.

**Figure 5:**
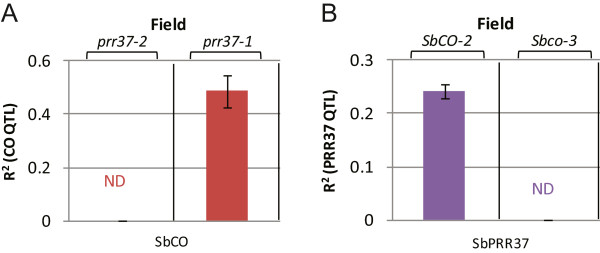
**Epistasis analysis of *SbPRR37 *and *SbCO *QTL in BTx642/Tx7000 RIL population under field conditions. A**. Proportion of phenotypic variance (R^2^) explained by the QTL corresponding to *SbCO-2*/*Sbco-3* in the portion of the population homozygous for *Sbprr37-1* (right) or *Sbprr37-2* (left). **B**. Proportion of the phenotypic variance explained by the QTL corresponding to *Sbprr37-1/Sbprr37-2* in the portion of the population homozygous for *SbCO-2* (left) or *Sbco-*3 (right). Each R^2^ value represents the average obtained under field conditions in three years.

If SbPRR37 delays flowering by inhibiting SbCO, and SbCO increases expression of *SbEhd1* and *SbCN8/12*, then epistatic interaction between SbPRR37 and SbCO may be detected in the RIL population. *SbPRR37* and *SbCO* allelic interactions were examined by first sorting the RILs into lines that contain *Sbprr37-1* (null) or *Sbprr37-2*, and then analyzing the influence of *SbCO* and *SbEHD1* alleles on flowering time in each background. In the portion of the population containing the null version of *Sbprr37-1*, the QTL corresponding to *SbCO*/*Sbco-3* (LOD = 13) explained 48% of the phenotypic variance for flowering time in the field (Figure 5A). In contrast, in the portion of the RIL population containing the active allele of *Ma1* (*Sbprr37-2*), no QTL corresponding to *SbCO* was observed. In this portion of the RIL population (*Sbprr37-2)*, the QTL corresponding to *SbEhd1-1*/*Sbehd1-2* explained ~20% of the phenotypic variance (date not shown). This result indicates that Sbprr37-2 inhibits SbCO-mediated induction of flowering. If this hypothesis is correct, then the ability of *Sbprr37-2* to inhibit flowering could be dependent on an active allele of *SbCO*. To test this hypothesis, the RIL population was sorted into lines that contained *SbCO-2* and lines that contained *Sbco-3*, and flowering time QTL were identified in each background (Figure 5B). This analysis showed that *SbPRR37* alleles affected flowering time in the *SbCO-2* background but not in the genetic background containing *Sbco-3* alleles, indicating that the ability of SbPRR37 to inhibit flowering is dependent on SbCO.

## Discussion

Sorghum accessions exhibit a wide range of flowering times when plants are grown in long days (i.e., 48d to >175d under field conditions in College Station, Texas) [2]. A large extent of this variation is caused by differences in photoperiod sensitivity mediated by floral repressors encoded by *Ma1* and *Ma6* that inhibit flowering in long days [1,29]. Much less is known about floral activators in sorghum. The grass specific floral activator *SbEHD1* was previously identified based on the gene’s sequence similarity to rice *EHD1* and activation of *SbEHD1* expression coincident with floral initiation [2]. In this study we identify and characterize a second activator of sorghum flowering *SbCO*, a homolog of the floral activator *CONSTANS* in Arabidopsis and an ortholog of *Hd1* in rice. Coding alleles of *CONSTANS* were identified through analysis of a flowering time QTL on SBI-10. Results showed that SbCO functions as an activator of flowering in LD and SD in sorghum genotypes using RILs with null versions of *Sbprr37-1* and *Sbghd7-1.* The *Sbco-3* allele in BTx642 was remarkable because it contained a His106Tyr amino acid substitution that also inactivates *CO* function in Arabidopsis [30]. Sorghum and Arabidopsis genotypes containing the inactive His106Tyr *co-3* allele flower late in long days, as well as late in short days in sorghum, indicating that CONSTANS functions as an activator of flowering in both species. SbCO shares a conserved CCT (CO, CO-like, TOC1) domain with TOC1, PRR37, Ghd7, and HEME ACTIVATOR PROTEINS (HAP or NF-Y proteins). Yeast two-hybrid screens showed that CO can interact with HAP3 and HAP5 subunits through its CCT-domain, forming CCAAT-binding CBF-complexes that bind to FT promoters and activate transcription [48,49]. In sorghum, SbCO was found to activate transcription of *SbEHD1*, *SbCN8* and *SbCN12*, consistent with its role as an activator of flowering, presumably through formation of CBF-complexes, but possibly through direct binding to DNA [50].

**Figure 6:**
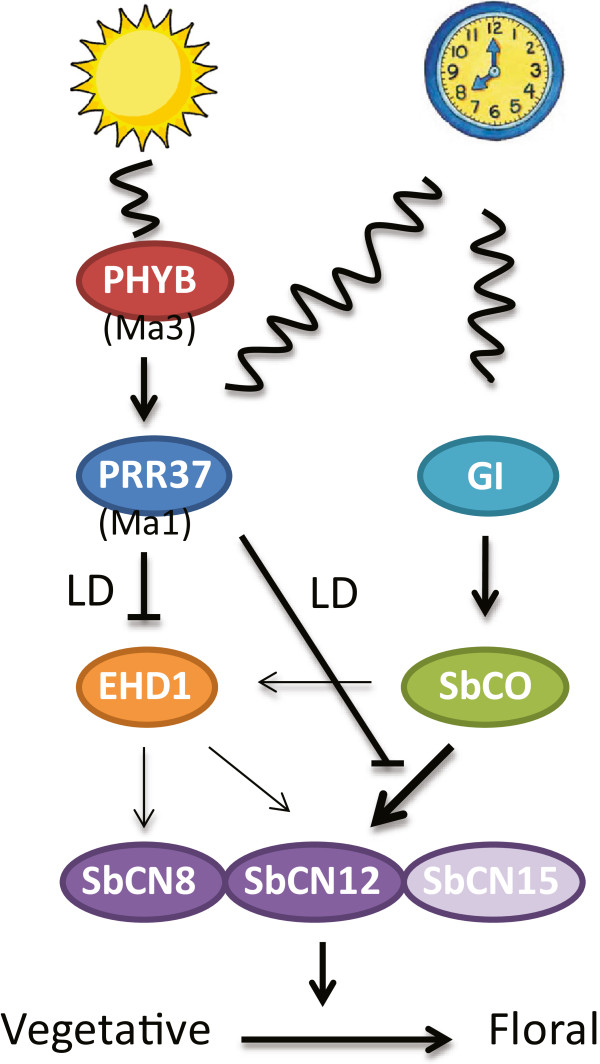
**Model of photoperiod flowering-time regulation in sorghum.** Phytochrome B mediates light signaling, providing information about day length required for photoperiod detection. Light and the circadian clock regulate expression of PRR37 resulting in higher expression in LD vs. SD. PRR37, a floral repressor, inhibits the activity of SbCO, a floral activator resulting in delayed flowering in LD. In inductive SD, SbCO activates expression of *SbEHD1*, *SbCN8* and *SbCN12* genes thereby promoting flowering. Thin lines represent possible mechanism of flowering time regulation.

The ability of *SbCO* alleles to induce flowering pathway gene expression and flowering was examined in RIL genetic backgrounds that contained null alleles of *Ma1* (*Sbprr37-1*) and *Ma6* (*Sbghd7)* to eliminate the influence of these LD floral repressors. In this null genetic background, SbCO promoted early flowering in LD and SD and increased the expression of *SbEHD1* (~25-fold), *SbCN8* (~10-fold), *SbCN12* (~100-fold) and *SbCN15* (~5-fold) relative to their expression in lines carrying the inactive *Sbco-3* allele. This information is summarized in a flowering time regulatory model shown in Figure 6. The model includes three members of the PEBP-gene family that could be sources of florigen in sorghum, *SbCN8*, *SbCN12* and *SbCN15. SbCN8* is an ortholog of maize *ZCN8*, with a pattern of gene expression consistent with the demonstrated role of *ZCN8* as a source of florigen in maize [47]. *SbCN12* expression is repressed by PRR37, induced in leaves in SD, and induced by *SbCO* (this study), indicating that this gene is also a likely source of florigen in sorghum. In rice, *Hd3a* and *RFT1* have been identified as sources of florigen; therefore expression of *SbCN15,* the ortholog of *Hd3a*, was analyzed. *SbCN15* showed relatively small changes in gene expression in response to photoperiod and mutations in SbPRR37 and SbCO. The sorghum genome does not encode an ortholog of *RFT1*, a source of florigen in rice in LD.

The results indicate that there has been a significant change in the complement of *FT*-like genes that function as the main sources of florigen in sorghum (*SbCN8*, *SbCN12*) and rice (*Hd3a* = *SbCN15*; *RFT1*, no sorghum ortholog), therefore regulation of flowering time could also differ, even though both grass species are short day plants. SbCO activates expression of *SbCN8* and *SbCN12*, although *SbCN12* was induced to a significantly greater extent. SbCO also increased expression of *SbEHD1*, an activator of *Hd3a* expression in rice. *SbEHD1* expression is repressed by SbPRR37 and SbGhd7 and induced when photoperiod sensitive sorghum grown in LD is transferred to SD [1,2]. Increases in *SbEHD1* expression occur in parallel with increases in *SbCN8* and *SbCN12* expression, suggesting that SbEhd1 can induce the expression of these genes as shown in Figure 6. However, the extent and specificity of this proposed activity of SbEhd1 will require further analysis in backgrounds where SbCO has minimal influence on the expression of these genes (*Sbco-3* backgrounds). The results in this paper show that SbCO increases expression of *SbEHD1* and it is proposed that SbEhd1 can activate expression of *SbCN8* and *SbCN12* and flowering. In contrast, rice Hd1 has not been reported to increase expression of *EHD1*[20,33]. Of interest is the finding that SbCO activates expression of *SbCN12* to a much greater extent than *SbEHD1.* Therefore we conclude that SbCO directly increases *SbCN12* transcription and that this may be the most important way that SbCO activates flowering.

The finding that SbCO can activate flowering in LD and SD in sorghum genotypes that are null for *Ma1* and *Ma6* raised the question as to how the activity of this gene is regulated by photoperiod in this short day plant. *SbCO* expression is low during the day, then increases in the evening to a peak at ~15 h after dawn, followed by a decrease and a second peak of expression at dawn (Figure 3B). In Arabidopsis, a similar increase in *CO* expression in the evening is due to the interaction of GI and blue light-activated FKF1, resulting in degradation of CDF-factors that inhibit *CO* expression [9]. This mechanism may also explain the evening peak of *SbCO* expression in sorghum. The second less prominent peak of *SbCO* expression at dawn is modulated by alleles of *SbPRR37* and enhanced in LD [1]. The function of the peak of *SbCO* expression at dawn is not currently understood, although production of SbCO at this time could help activate *SbEHD1* expression in the morning.

Functional alleles of *SbCO* increased the expression of *SbCN8* and *SbCN12* to a greater extent in SD relative to LD (Figure 4). SbCO expression levels increase during the evening, helping to explain why *SbCN8/12* expression increases at night. Since expression of *SbCO* was not altered significantly by photoperiod (Additional file 5: Figure S3), increased activity of SbCO in SD is most likely due to an increase in protein level or activity. In Arabidopsis, a long day plant, CO levels are higher in LD due to COP1-SPA1-Cry2 stabilization of the protein [6]. This stabilization module may be missing or attenuated in sorghum. Reduced PhyB/C-mediated degradation of SbCO in SD, relative to LD, could result in greater SbCO-mediated activation of SbCN8/12 in SD. In sorghum genotypes containing active alleles of *SbPRR37* the evening peak of *SbCO* expression is not altered, the peak of expression at dawn increases, but the activity of SbCO is strongly attenuated. Expression of *SbPRR37* is high in the evening in plants grown in LD, but low in SD. Therefore, higher levels of *SbPRR37* expression in LD, and SbPRR37 repression of SbCO activity under these conditions, is predicted to prevent SbCO from activating flowering in LD.

SbPRR37 is a CCT-domain protein that has been shown to interact with HAP3, the same CBF-subunit that interacts with SbCO [51]. Therefore, SbPRR37 may be a competitive inhibitor of SbCO binding to the HAP complex. SbPRR37 may also directly bind to DNA in a fashion similar to TOC1 and other PRR-proteins [52]. TOC1 binding to its cognate motif in the promoter of LHY/CCA1 is mediated by its CCT-domain, resulting in PRR-domain mediated repression of transcription. If PRR37 binds to the *SbCN12* promoter in a similar manner, it could directly repress transcription, block SbCO binding to the HAP complex, and/or interact with CO or other proteins in order to repress *SbCN12* transcription. Recent results on the PRR37 ortholog in rice (Hd2) indicated that PRR37 directly represses *Hd3a* transcription [35]. Further genetic and biochemical analysis will be required to distinguish among these possibilities.

In barley, a long day plant, HvCO1 activates flowering in LD, and activation is dependent on Ppd-H1, an ortholog of SbPRR37. Overexpression of *HvCO1* induced flowering in both LD and SD, but photoperiod sensitivity mediated by *Ppd-H1* was still observed in this background [36]. Ppd-H1 does not directly affect expression of *HvCO1*, but potentiates the ability of HvCO1 to activate *HvFT1* expression in LD. It is interesting to note that Ppd-H1 increases HvCO1 activity in LD, whereas SbPRR37 inhibits the floral promoting activity of SbCO in LD. The expression and activity of SbPRR37 and Ppd-H1 increase in LD and both affect CO’s ability to modulate FT-gene expression, but in an opposite manner, consistent with barley being a long day plant and sorghum a short day plant. The difference in activity of PRR37 could be due to differences in direct binding of PRR37 to the promoters of *SbCN12* and *HvFT1* (homolog of *Hd3a*, *SbCN15*), or indirectly by interaction of PRR37/Ppd-H1 with activators, repressors, HAP subunits, HvCO1 and SbCO. In rice, it has been suggested that phosphorylation of PRR37 by Hd6 may cause PRR37, in conjunction with Hd1, to become a repressor of *Hd3a* expression in LD [35]. The possibility that PRR37 can form a co-repression complex with CO is consistent with results in sorghum and rice. However in the absence of PRR37, CO functions as an activator of *FT* expression in sorghum and rice. While the biochemical basis of variation in PRR37 activity remains to be elucidated, taken together, the results suggest that interaction between PRR37 and CO on the promoters of specific florigen-related PEBP-genes result in fundamental differences in photoperiod-sensitive flowering between LD and SD grasses.

The flowering time model in Figure 6 shows that SbEhd1 and SbCO can independently induce flowering by activating *SbCN8* and/or *SbCN12.* EHD1 and Hd1 have been shown to independently activate Hd3a (FT) and flowering in rice [20]. In sorghum we show that there is cross-talk between these pathways because SbPRR37 activates *SbCO* expression at dawn in LD, while SbCO induces *SbEHD1* expression in SD. Rice EHD1, a B-type response regulator, is controlled by several upstream modulators including the repressors GHD7, GRAIN NUMBER, PLANT HEIGHT AND HEADING DATE 8 (GHD8), OsLEC1 and FUSCA-LIKE1 (OsLFL1), OsMADS56 and the activators GI, EARLY HEADING DATE 2 (EHD2) and OsMADS50 [8,53]. The existence of two parallel pathways that can activate flowering in sorghum provides for a wide range of responses to diverse environmental factors, contributing to sorghum’s wide geographical adaptation. Sorghum crop breeders are utilizing different alleles of key genes in these parallel pathways to generate early flowering grain sorghum hybrids and late flowering energy sorghum hybrids.

## Conclusions

Alleles of sorghum *CONSTANS* (*SbCO*) were identified through analysis of a flowering time QTL on chromosome 10 identified in a RIL population derived from BTx642 and Tx7000. Genetic analysis and gene expression studies indicate that SbCO is an activator of flowering in long and short days in genotypes lacking active SbPRR37 and GHD7 alleles. PRR37 was found to block CO-mediated floral activation in long days.

## Methods

### Plant materials

The BTx642/Tx7000 RIL population (n = 90) and parental lines were grown under field conditions in a replicated randomized block design at Texas A&M Research Farm near College Station Texas in 2008, 2009 and 2010 with planting between April 1–14. Days to mid-anthesis (pollen shed) were determined as a measure of flowering time. In the field, day-lengths increased from ~12.6 h in April to 14.3 h in July, with an average daily maximum temperature of 31.7°C and an average daily minimum temperature of 20.0°C. Ten plants of each RIL and the parental lines were grown in a greenhouse in 10 h day lengths (SD, 2011) or 14 h day lengths (LD, 2009 and 2010) and phenotyped for flowering time in a similar manner as the populations grown in the field. RIL105 and RIL112 correspond to 4_6 and 12_14 in original BTx642/Tx7000 RIL population [38].

### Genotyping by sequencing and QTL analysis

Genotyping by sequencing was carried out using Digital Genotyping (DG) [40] on the 90 RILs derived from BTx642 and Tx7000 [39]. A genetic linkage map was constructed using data generated from 1462 polymorphic DG markers using Mapmaker/EXP ver. 3.0b where recombination frequency was calculated using the Kosambi mapping function. QTLs were detected using Composite Interval Mapping (CIM) in WinQTL Cartographer v2.5 [54]. Significant LOD thresholds for QTL detection were determined based on experiment-specific permutations with 1000 repeats at α = 0.05 [55]. In QTL-based epistasis analysis, the 90 RILs were categorized into sub-populations based on alleles of *SbPRR37* or alleles of *SbCO* respectively. Sub-populations homozygous for each allele of *SbPRR37* and each allele of *SbCO* were then subjected to QTL analysis.

### Phylogenetic and Colinearity Analysis

The amino acid sequence of rice Hd1(Os06g16370) was used to search Phytozome v9.1 [42] for homologs of CONSTANS in rice, maize, barley and sorghum. Multiple sequence alignment, alignment scores and phylogentic analysis were performed using ClustalW2 [56] using protein sequences of Sb10g010050 (Sorghum CO), GRMZM2G405368_T01 (Maize conz1), Os06g16370 (Rice Hd1), AF490468 (Barley HvCO1) and AT5G15850 (Arabidopsis CO). Rice and sorghum genome sequences (Phytozome v9.1, 100 kbp) spanning homologs of *CO* were used for synteny/colinearity analysis. Colinearity was determined by GEvo [44], a high-resolution sequence analysis tool of genomic regions from CoGe (Accelerating Comparative Genomics) tool kit [57]. A similar phylogenetic/colinearity analysis was performed for *EHD1*.

### Allele characterization

SNPs in candidate genes were identified by comparing DNA sequences derived from BTx623, BTx642 and Tx7000. The BTx623 *Sbi1* assembly and *Sbi1.4* gene annotation were used as the reference genome sequence (Phytozome). BTx642 and Tx7000 genome sequence assemblies used for analysis were obtained previously [39]. SNPs were called using the SNP Detection function of CLC Genomics Workbench 4.9. Minimum coverage for a variant call was set at 5, and maximum was set at 150. Allele types were designated based on SNPs. The SIFT algorithm (sorting intolerant from tolerant) [45] was utilized to predict whether an amino acid substitution affects protein function based on the degree of conservation of amino acid residues in sequence alignments derived from closely related gene sequences. RIL105 and RIL112 were selected from the BTx642/Tx7000 RIL population using DG markers flanking each QTL peak and spanning each candidate gene. RIL112 contains BTx642 haplotypes spanning all of the flowering time QTL, including the QTL on SBI-10 (*Sbco-3*). RIL105 contains BTx642 haplotypes spanning QTL on SBI-01, SBI-06, SBI-08 and the Tx7000 haplotype spanning the QTL on SBI-10 (*SbCO-2*) (Additional file 6: Table S3).

### LD, SD and circadian experiments

For circadian rhythm experiments, RIL105 and RIL112 were grown in the greenhouse in LD (14 h light) for 32 days. For entrainment, the plants were transferred to growth chambers set for LD (14 h light/10 h dark) or SD (10 h light/14 h dark) treatment for one week prior to collection of tissue for expression analysis. In the growth chamber, daytime temperature was 30°C at a light intensity of ~300 μmol · s^−1^ · m^−2^ and night (dark) temperature was 23°C with ~60% relative humidity. At day 39, the fully expanded portion of the top three leaves from three different plants were sampled from each genotype and photoperiod every 3 hours through a 24 h light–dark cycle followed by 24 h of continuous light (continuous 30°C) (LL). RNA was extracted from leaf tissues using TRI Reagent (MRC) using the protocol for tissues with high polysaccharide content. RNA was cleaned up using RNeasy Mini Kits (QIAGEN), including DNA removal by on-column DNase I digestion. RNA integrity was examined on 1% MOPS gels. First-strand cDNA synthesis was performed using the SuperScript® III First-Strand Synthesis System (Invitrogen) with oligo dT and random hexamer primer mix. After first-strand cDNA synthesis, the reactions were diluted to a final concentration of 10 ng/μl of the initial total RNA. Gene-specific qRT-PCR was performed using Power SYBR Green PCR Master Mix (Applied Biosystems). 18S rRNA was selected as internal control and detected using the TaqMan Universal PCR Master Mix (Applied Biosystems), rRNA Probe (VIC® Probe) and rRNA Forward/Reverse Primer. All reactions were run on a 7900HT PCR System with SDS v2.3 software (Applied Biosystems). The specificity of each qRT-PCR primer set was validated using melting temperature curve analysis. Amplification efficiency of each primer set was determined by the series dilution method [58], which can be calculated by the slope of the curve made from each Ct value and the dilution factor (Additional file 7: Table S4). Relative expression was determined using the comparative cycle threshold (ΔΔCt) method with calibration using samples with the highest levels of RNA. The primer efficiency was employed to adjust data for relative quantification following the efficiency correction method [59]. Each expression data point was derived from analysis of three technical replicates within three biological replicates.

### Availability of supporting data

All the supporting data are included as additional files. BTx642 and Tx7000 whole genome sequence assemblies used for analysis were published previously in Evans J, McCormick RF, Morishige D, Olson SN, Weers B, et al. (2013) Extensive Variation in the Density and Distribution of DNA Polymorphism in Sorghum Genomes. PLoS ONE 8(11): e79192. doi:10.1371/journal.pone.0079192. Genome sequences were deposited with NCBI SRA under the following identifiers: BioProjectPRJNA189453, Accession SRP019171 (http://www.ncbi.nlm.nih.gov/sra/?term=PRJNA189453); SAMN01942195 contains sequence information for BTx642, and SAMN01942194 contains sequence information for Tx7000.

## Competing interests

The authors declare that they do not have competing interests.

## Authors’ contributions

SY contributed to the design of experiments, collected and analyzed data, and was primarily responsible for figure construction and writing the paper. JM conceived and obtained funding for the research, helped design and interpret experimental results and edited the text. BW phenotyped the RIL population for flowering time in multiple locations and conducted QTL analysis in collaboration with SY. DM helped to identify the nature of CO alleles and other flowering time gene alleles, and aided in the design of experiments, collection of tissue used to analyze flowering gene expression and editing of the text. All authors read and approved the final manuscript.

## Supplementary Material

Additional file 1: Table S1 Characterization of *SbEHD1* alleles. Click here for file

Additional file 2: Figure S1 Colinearity of rice Hd1 and sorghum CONSTANS. Reference genome sequences including sorghum *SbCO* (upper panel) and rice *OsHd1* (lower panel) were analyzed for sequences that align (red boxes) Colinear genes within the aligned region are connected by red lines. a-d represent four colinear genes in rice and sorghum (Sb10g010020- Sb10g010050) including *SbCO* (Sb10g010050, d). Click here for file

Additional file 3: Figure S2 Expression level (ΔCt) of circadian clock genes and GI in RIL105 (black solid line) and RIL112 (red dashed line) under either LD (14 h light/10 h dark) or SD (10 h light/14 h dark) conditions. The gray shaded area represents the dark period. The first 24 h covers one light–dark cycle, followed by 24 h of continuous light. A. TOC1. B. LHY. C. GI. Each expression data point corresponds to three technical replicates within three biological replicates. Click here for file

Additional file 4: Table S2 Orthologs of sorghum *FT/CN* genes (*SbCN*) identified in maize and rice. Click here for file

Additional file 5: Figure S3 Relative expression levels of *SbCO* in RIL105 grown in LD (14 h light/10 h dark) or SD (10 h light/14 h dark). Black solid lines represent relative expression in LD and red dashed lines represent relative expression in SD followed by 24 h in LL. Click here for file

Additional file 6: Table S3 Genotypes of genetic loci affecting flowering time in RIL112 and RIL105. Click here for file

Additional file 7: Table S4 Primer sequences and amplification efficiency of primers used for qRT-PCR. Click here for file
