# Supplementary figures and images for: CONSTANS is a photoperiod regulated activator of flowering in sorghum
Source: BMC Plant Biol. 2014 May 28;14:148. doi: 10.1186/1471-2229-14-148 (PMC4046011; doi:10.1186/1471-2229-14-148)

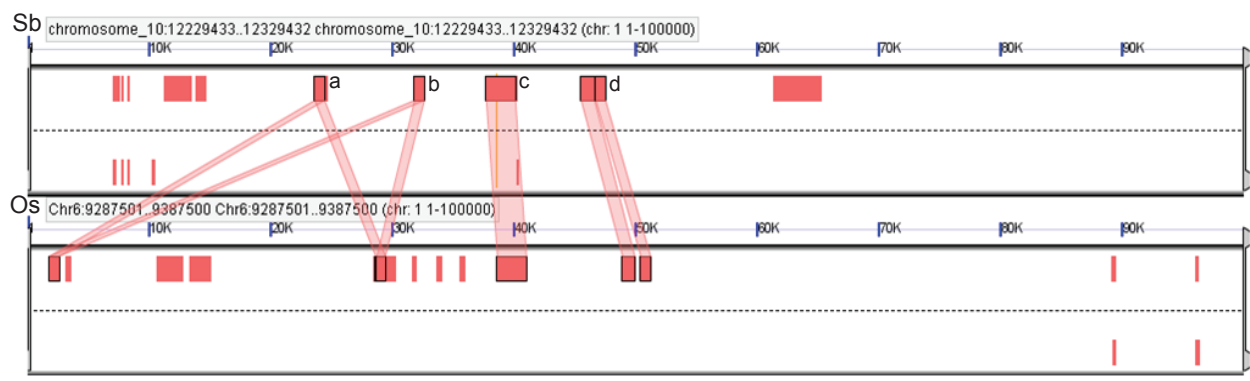

Supplement: Additional file 2: Figure S1 — Colinearity of rice Hd1 and sorghum CONSTANS. Reference genome sequences including sorghum SbCO (upper panel) and rice OsHd1 (lower panel) were analyzed for sequences that align (red boxes) Colinear genes within the aligned region are connected by red lines. a-d represent four colinear genes in rice and sorghum (Sb10g010020- Sb10g010050) including SbCO (Sb10g010050, d). [file 1471-2229-14-148-S2.pdf]

A

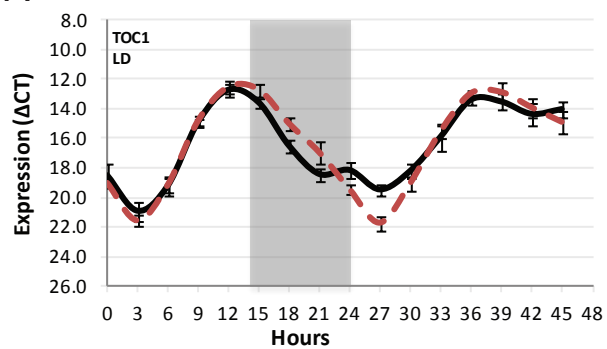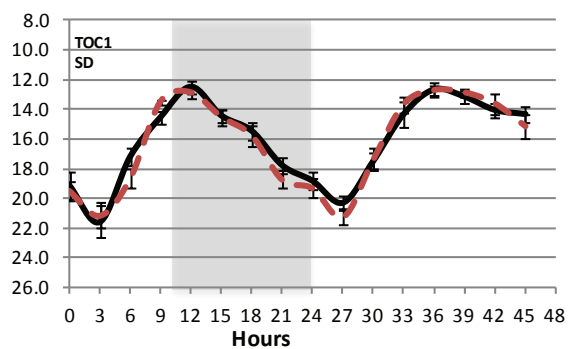

B

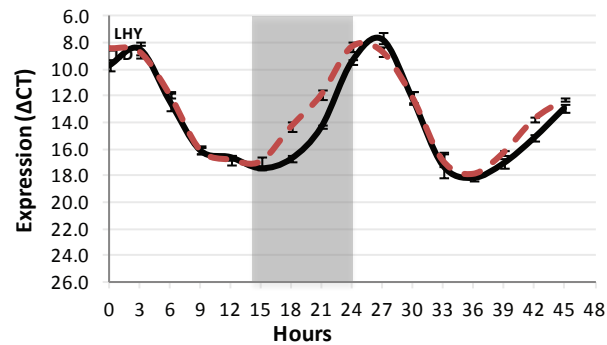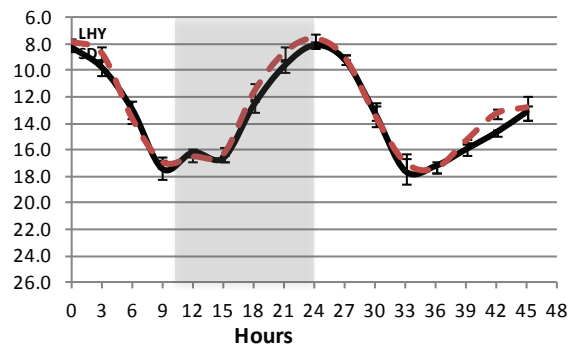

C

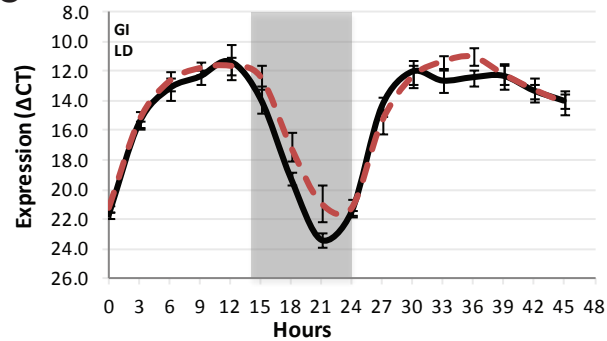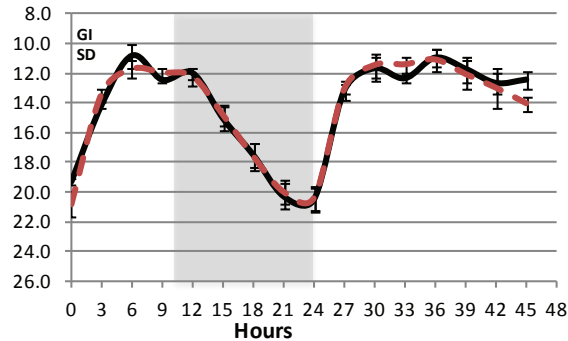

Supplement: Additional file 3: Figure S2 — Expression level (ΔCt) of circadian clock genes and GI in RIL105 (black solid line) and RIL112 (red dashed line) under either LD (14 h light/10 h dark) or SD (10 h light/14 h dark) conditions. The gray shaded area represents the dark period. The first 24 h covers one light–dark cycle, followed by 24 h of continuous light. A. TOC1. B. LHY. C. GI. Each expression data point corresponds to three technical replicates within three biological replicates. [file 1471-2229-14-148-S3.pdf]

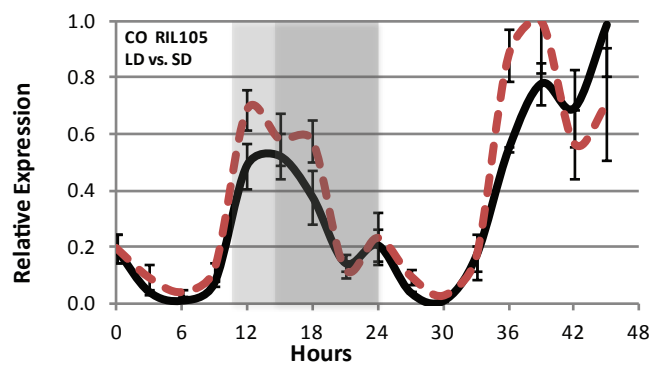

Supplement: Additional file 5: Figure S3 — Relative expression levels of SbCO in RIL105 grown in LD (14 h light/10 h dark) or SD (10 h light/14 h dark). Black solid lines represent relative expression in LD and red dashed lines represent relative expression in SD followed by 24 h in LL. [file 1471-2229-14-148-S5.pdf]
