# Supplementary material for: CONSTANS is a photoperiod regulated activator of flowering in sorghum
Source: BMC Plant Biol. 2014 May 28;14:148. doi: 10.1186/1471-2229-14-148 (PMC4046011; doi:10.1186/1471-2229-14-148)
Supplement: Additional file 4: Table S2 — Orthologs of sorghum FT/CN genes (SbCN) identified in maize and rice. [file 1471-2229-14-148-S4.pdf]

Table S2. Orthologs of sorghum *FT/CN* genes (*SbCN*) identified in maize and rice.

Sorghum Gene	Locus ID in Sorghum*	Location	Maize Gene	Locus ID in Maize	Rice Gene	Locus ID in Rice
SbCN8	Sb09g025760	chr_9: 55149243 - 55150558	*ZCN8*	GRMZM2G179264	*FTL10*	LOC_Os05g44180
SbCN12	Sb03g034580	chr_3: 62753997 - 62755638	*ZCN12*	GRMZM2G103666	*FTL9*	LOC_Os01g54490
SbCN15	Sb10g003940	chr_10: 3464074 - 3465644	*ZCN15*	GRMZM2G051338	*FTL2 (Hd3a)*	LOC_Os06g06320

* Gene Locus IDs in Sorghum, Maize and Rice are from Phytozome v9.1 (http://www.phytozome.net/).
