# Supplementary material for: CONSTANS is a photoperiod regulated activator of flowering in sorghum
Source: BMC Plant Biol. 2014 May 28;14:148. doi: 10.1186/1471-2229-14-148 (PMC4046011; doi:10.1186/1471-2229-14-148)
Supplement: Additional file 6: Table S3 — Genotypes of genetic loci affecting flowering time in RIL112 and RIL105. [file 1471-2229-14-148-S6.pdf]

Table S3. Genotypes of genetic loci affecting flowering time in RIL112 and RIL105.

FLW Locus (QTL)	Candidate Gene	RIL112 (allele origin)	RIL112 (allele type)	RIL105 (allele origin)	RIL105 (allele type)
Chr_01	*EHD1*	BTx642	*Sbehd1-2*	BTx642	*Sbehd1-2*
Chr_06	*PRR37*	BTx642	*Sbprr37-1*	BTx642	*Sbprr37-1*
Chr_06	*GHD7*	BTx642	*Sbghd7-1*	BTx642	*Sbghd7-1*
Chr_08	ND*	BTx642	active	BTx642	active
Chr_10	*SbCO*	BTx642	*Sbco-3*	Tx7000	*SbCO-2*

* ND: Not determined.
