# Supplementary material for: CONSTANS is a photoperiod regulated activator of flowering in sorghum
Source: BMC Plant Biol. 2014 May 28;14:148. doi: 10.1186/1471-2229-14-148 (PMC4046011; doi:10.1186/1471-2229-14-148)
Supplement: Additional file 7: Table S4 — Primer sequences and amplification efficiency of primers used for qRT-PCR. [file 1471-2229-14-148-S7.pdf]

Table S4. Primer sequences and amplification efficiency of primers used for qRT-PCR.

Gene	Locus ID in Sorghum*	Forward Primer	Reverse Primer	Amplification Efficiency in RIL105	Amplification Efficiency in RIL112
SbCO	Sb10g010050	CGGTACGGTTCTATGGTTCTG	AACTACTTGTACACTCGACCTTATC	0.82	0.79
SbEHD1	Sb01g019980	CGTCAGGGAAGCAATGTCCTTCAT	CTTCAGTTGGAAAGCACACATCGC	0.88	0.81
SbCN8	Sb09g025760	AACTGTCAAAGGGAAGGTGGATCG	GACTAAGCTCTCAACCCTTCAAGTC	0.85	0.84
SbCN12	Sb03g034580	TGCATGCATGAATATCGTCGTCT	CCCGGGTAGTACATATAAGGTGGT	1.03	0.91
SbCN15	Sb10g003940	GCTAGCTTATCCCGCATATTACCC	CCACCCAAACTGCATCCACTCTTGAA	0.83	0.80
SbGI	Sb03g003650	ATGCACCCGCTTCCTAGTCATCTT	TTCAGGGCTGTCATGGTTCCTCAT	0.85	0.84
SbTOC1	Sb04g026190	GAGTGCAGATGATTACTGCTCACTTTG	TGCTGCCTTGTTGCCAGTAGAAGA	0.78	0.79
SbLHY	Sb07g003870	GGCCTGCCTCTACCATGAAGTTTA	GCACTGCATTGCAAGGTTTGAAGTCC	0.81	0.82

* Gene Locus IDs in Sorghum are from Phytozome v9.1 (http://www.phytozome.net/).
