# Supplementary material for: CONSTANS is a photoperiod regulated activator of flowering in sorghum
Source: BMC Plant Biol. 2014 May 28;14:148. doi: 10.1186/1471-2229-14-148 (PMC4046011; doi:10.1186/1471-2229-14-148)
Supplement: Additional file 1: Table S1 — Characterization of SbEHD1 alleles. [file 1471-2229-14-148-S1.pdf]

Table S1. Characterization of *SbEHD1* alleles.

SNP #	1	2	3	4	5	6	7	8	9
Location (SBI-01)	21921341	21922009	21922977	21923172	21923861	21923883	21924404	21924444	21925032
Nucleotide Variation	G>A	C>G	G>A	C>T	T>C	C>T	G>A	C>T	G>A
Protein Modification	No change	Intron	Intron	Intron	Intron	Intron	Asp144Asn	Thr157Ile	Intron
EHD1 Domain							GARP	GARP	
***SbEhd1-1* (Tx7000, BTx623)**	-	-	-	-	-	-	-	-	-
***Sbehd1-2* (BTx642)**	+	+	+	+	+	+	+	+	+
